# A comparative study of online communities and popularity of BBS in four Chinese universities

**DOI:** 10.1371/journal.pone.0234469

**Published:** 2020-06-24

**Authors:** Hao-Nan Yang, Xin-Jian Xu, Haili Liang, Xiaofan Wang

**Affiliations:** 1 Department of Mathematics, Shanghai University, Shanghai, China; 2 School of Mechatronic Engineering and Automation, Shanghai University, Shanghai, China; Beijing University of Posts and Telecommunications, CHINA

## Abstract

Online forums in Chinese universities play an important role in understanding collective behavior of college students. Of particular interest are community and popularity. We address these two issues by examining data from Bulletin Board Systems (BBSs) of four Chinese universities. To characterize users’ behavior, we introduce a hypothesis test to infer individual preferred boards, which yields a polarization of users. We also perform a multilevel algorithm to detect communities of each BBS network. We measure the similarity between the board-preferred polarization and the algorithmically identified community structure by quantitative and visual tools. The resulting discrepancy indicates that board labels are inadequate to represent underlying communities. To reveal online popularity, we employ latent Dirichlet allocation to mine topics from threads to compare popularity in different universities. Based on which, we implement the Cox-Stuart test to explore the change in popularity over time and reproduce significantly ascending and descending topics around a decade. Finally, we devise a two-step model based on users’ preference and interests to reproduce the observed connectivity patterns.

## Introduction

College life is important for adolescents because it is not only the first experience independent of their parents but also a crucial stage of the formation of worldview. In China, most college students live without high pressure and develop their interests freely. It therefore is important to understand what they are interested in and how their interests evolve. Over the last two decade, web 2.0 technologies boosted new forms of communication and produced big data, allowing us to study population behavior at unprecedented levels of size and detail [1]. For instance, online social networks, such as Facebook and Twitter, have attracted hundreds of millions of users, especially young people [2–4]. Whereas for Chinese college students, they mainly used campus BBSs. Each Chinese university has a local BBS on which users can share interests, express opinions, discuss about collegiate life and national affairs. They communicate by creating and replying threads in hundreds of discussion boards (sub-forums) based on personal interests. Analyzing this time-stamped, unstructured knowledge repository could provide key insights about collegiate communities and popularity.

One way to visualize and extract core information of individual behavior is employing concepts from complex network theory [5]. Regarding creating and replying relationships in BBSs as interactions among users, several papers have attempted to understand online collegiate networks. Zhongbao and Changshui [6] selected six boards from the BBS at Tsinghua University, China. They utilized articles posted between October and December in 2001 to build reply networks, and analysed the degree distribution, clustering coefficient and shortest path length. Goh et al. [7] considered all the threads posted from March 2000 to November 2004 from the BBS at Korea Advanced Institute of Science and Technology. They examined separately the degree distribution of the student network and the size distribution of the board network. On the contrary, Panzarasa et al. [8] studied longitudinal characteristics of an online community at University of California, Irvine. With the data covers the period from April to October in 2004, they not only investigated temporal evolution of the nodal degree, clustering coefficient and giant component, but also compared the interevent time distributions for single users, discussion groups, and the whole forum to examine temporal correlations and bursty patterns of communication [9].

The focus of these studies remained primarily either on the level of single individuals or on the level of the whole system. What is still largely to be investigated is the meso level. In a BBS, users polarize with specific interests, hence the formation of communities [10]. In this way, users in the same community are highly connected, while there are few links among the users belonging to different communities. Detection of these communities may help us to identify functional units such as topics in information systems [11], which reflect common interests of college students. To compare algorithmically obtained communities to partitions based on the given categorical data, Traud et al. [12] adopted pair counting and Rand coefficient as the similarity measure for Facebook networks of five U.S. universities at a single-time snapshot in September 2005. They found that the class year is the dominant attribute to community formation in the global view. Furthermore, Sung et al. [13] extracted the dominant attribute contributing to the local community. Nevertheless, both the studies didn’t investigate temporal characteristics of the data.

Considering latitudinal and longitudinal aspects of BBSs, we ask two questions: i) how college students form online “communities”in a BBS and what characteristics do these communities have? and ii) what popular topics appear in online collegiate communities and how does “popularity”evolve over time? To answer these questions, we examine data from four BBSs of Chinese universities around a decade. First, we introduce a null model [14] to infer users’ preferred boards based on their interests. Second, we analyse BBS networks whose links represent replying relationships between users (nodes). We carry out a multilevel algorithm [15] to identify communities of BBS networks and compare to polarized groups according to preferred boards. Then, we adopt latent Dirichlet allocation (LDA) [16] to automatically mine topics from text corpora of four BBBs, based on which we explore the trends of popular topics over time by the Cox-Stuart test [17]. Finally, we propose a simple model to reproduce the observed dynamics.

## Materials and Methods

### Data collection

We use web crawlers to download the data from four university official BBS forums: Wei Ming BBS (http://bbs.pku.edu.cn), Tian Di Ren Da BBS (http://bbs.ruc.edu.cn), Le Hu BBS (http://bbs.shu.edu.cn) and Ri Yue Guang Hua BBS (http://bbs.fdu.edu.cn). We crawled the data in accordance with these websites’ terms of services. We extract the content in particular HTML tags, including post ID, board, time stamp, replied ID. As for text content in message stream, we remove very common Chinese-language stop words such as *yi ge* (which means “a/an”in English). In addition, we remove some university-related words such *bei da* (“Peking University”in Chinese) and specific set of jargon in BBS, which do not help to create meaningful topics. The source data are available at https://www.kaggle.com/bbschn/bbsdata.

### Null model for preferred boards

It is based on the following hypothesis: supposing the total number of boards a user participating in is *m*, the normalized activities of boards are produced by a random assignment from a uniform distribution. One can implement this process by setting *m* − 1 uniform random points in the interval [0, 1] so that the interval is divided into *m* subintervals. Their lengths represent expected values of *m* normalized activities *a*_*i*_ corresponds to the user. The probability density function for one of the variables taking particular value *x* is [14]

$$\begin{array}{c} \rho \left(x\right)=\left(m-1\right){\left(1-x\right)}^{m-2}, \end{array}$$ (1)

which depends on *m* boards that users are involved in their lifetime span. The null model calculates the probability to determine whether there is evidence to reject the null hypothesis, known as *p*-value. In statistical inference, this concept is a probability that, if the null hypothesis is true, one obtains a value for the variable equal to or more extreme than the observed one. Noting that the function (1) is monotonically decreasing, “more extreme”can mean larger than the observed one.

### Empirical reply networks

In a BBS, users communicate by replying articles. Construction of a reply network is straightforward. All the user IDs, corresponding to college students, can be represented by nodes. A link is established between two nodes if they have a replying relationship in a article. In most cases, the replying relationships are reciprocal, so we ignore the directness of the link. The number of times they communicate with each other can be denoted by the weights of the link. After examining all the articles, an undirected and weighted network is constructed. We consider only ties among users at the same university, which yields four separate time-aggregated reply networks and allows us to compare the structural diversity of different universities in the same period 2006-2012.

### Community detection algorithm

The community detection algorithm is used to identify highly connected groups of nodes in a network. One metric to evaluate the the quality of the partition is so-called modularity, defined as a value between −1 and 1 that measures the density of links inside communities compared to links between communities. Here, we adopt the widespread Louvain method [15] to maximize modularity, the computational time of which is linear with number of links. The method consists of repeated execution of two steps: the first step is a greedy assignment for local optimizations of modularity and the second step is the definition of a new coarse-grained network based on the communities found in the first step. These two steps are repeated until no further modularity-increasing. The algorithm is simple, efficient and easy-to-implement for identifying communities in large networks.

### Rand index and its adjusted version

The Rand Index computes the similarity between two data clusterings. Given two kinds of classifications *P*_*a*_ and *P*_*b*_ for *n* nodes, we denote the count of node pairs that classified together in both partitions by *w*_11_, classified together in *P*_*a*_ but different in *P*_*b*_ by *w*_10_, different in *P*_*a*_ but classified together in *P*_*b*_ by *w*_01_ and different in both by *w*_00_. Noting that $w_{11}+w_{10}+w_{01}+w_{00}=C_{n}^{2}=M$, the Rand index can be defined by [18]

$$\begin{array}{c} S_{R}=\frac{w_{11}+w_{00}}{M}, \end{array}$$ (2)

which counts the fraction of pairs that are assigned in the same or different clusters both in *P*_*a*_ and *P*_*b*_, hence lying the interval [0, 1]. A problem with the Rand index is that its expected value between two random partitions is not a constant, but depends on the number *n* of nodes. Vinh et al. [19] proposed the adjusted Rand index which assumes that the randomness is generated by the hyper-geometric distribution,

$$\begin{array}{c} S_{\text{AR}}=\frac{w_{11}-\frac{1}{M}(w_{11}+w_{10})(w_{11}+w_{01})}{\frac{1}{2}[(w_{11}+w_{10})+(w_{11}+w_{01})]-\frac{1}{M}(w_{11}+w_{10})(w_{11}+w_{01})} \end{array}$$ (3)

Thus, *S*_AR_ ∈ [−1, 1] is the corrected-for-chance version of *S*_R_.

### LDA

The LDA is a generative statistical model, in which each document is characterized by a probability distribution over topics and each topic is in turn characterized by a probability distribution over words. Here, we use a novel unified topic modeling framework called Familia [20], which contains well-trained topic models based on various types of large-scale Chinese corpora, such as news, webpage, novel and Sina weibo (a Chinese microblogging website). The vocabulary table contains 294,657 Chinese words, and the preset topic size is 2,000 in LDA implementation. One of a user-specified parameter, which denoted as *k*, is the number of topics. The preset topic contain much redundancy sometimes. For any two topics *T*_1_ and *T*_2_, we consider the first *m* words and use the Jaccard similarity to evaluate the redundancy between the two topics,

$$\begin{array}{c} J\left(T_{1},T_{2}\right)=\frac{|T_{1}\cap T_{2}|}{|T_{1}\cup T_{2}|}=\frac{|T_{1}\cap T_{2}|}{|T_{1}\left|+\right|T_{2}\left|-\right|T_{1}\cap T_{2}|}, \end{array}$$ (4)

where |*T*| denotes the number of words in topic *T*. We define the threshold value *J*_0_ and if *J*(*A*_1_, *A*_2_) ≥ *J*_0_, the two topics have redundancy. Considering each topic as a node, each two nodes have a link if they have redundancy. For each connected component in this topic network, we can merge them into one topic. The number of refined topics equal to the number of the connected component. In this way, we set *m* = 10 and *J*_0_ = 0.01, under which 2, 000 topics are merged into 476 topics finally.

### Cox-Stuart test

The Cox-Stuart test is applied to assess whether there is an increasing or decreasing trend in independent time series, which is applicable to a wide variety of situations [17]. The statistical hypotheses in testing for trend in a series of random variables are: *H*_0_ (no monotonic trend exists in the series) and *H*_1_ (the series have an increasing or decreasing trend). Given a series of data *x*_1_, ⋯, *x*_*k*_, the Cox-Stuart test divide the series into two parts: *x*_1_, ⋯, *x*_*k*/2_ and *x*_*k*/2+1_, ⋯, *x*_*k*_. If *k* is odd, remove *x*_(*k*+1)/2_ and divided equally into two parts and set *k* ≔ *k* − 1. Then we obtain *k*/2 pairs: (*x*_*i*_, *x*_*i*+*k*/2_) for *i* = 1, ⋯, *k*/2. We define *T* as the number of pairs in which satisfy *x*_*i*_ < *x*_*i*+*k*/2_, i.e.,

$$\begin{array}{c} T_{0}=\sum_{i=1}^{k/2}1_{\left\{x_{i}<x_{i+k/2}\right\}}. \end{array}$$ (5)

If *T*_0_ > *k*/2 − *T*_0_, we have more pairs with upward trend than downward trend, the statistic *T* = *T*_0_ for testing the ascending trend. Otherwise *T* = *k*/2 − *T*_0_ which tests the descending trend. If the null hypothesis *H*_0_ is true, the statistic *T* follows the binomial distribution with parameters *k*/2 and 1/2, i.e., *T* ∼ *B*(*k*/2, 1/2). So the *p*-value is

$$\begin{array}{c} p=\sum_{i=T}^{k/2}(\begin{array}{c} n\\ i \end{array}){\left(\frac{1}{2}\right)}^{i}{\left(1-\frac{1}{2}\right)}^{k/2-i} \end{array}$$ (6)

Imposing the significance level *α*, the trend that satisfy *p* < *α* can be determined whether it is ascending or descending.

## Results

Campus BBSs of Chinese universities, retrospective to the later 1990s, are most active and prevalent cyberspace in universities. Billions of articles have been posted by millions of college students, which record student interests and collegiate culture. A BBS has a hierarchical (tree-like) structure: the BBS site contains hundreds of boards, each of which was categorized by special topics. Within a forum’s board, each new discussion is called a thread created by an initial article and followed by reply articles (see S1 Fig). Different from online forums in other countries, Chinese campus BBSs have two key properties guaranteeing them as a good data resource for present research [21]. One is the registration rule. Each campus BBS only allows enrolled students to sign up with their student IDs. Thus, all the articles were created and replied by college students, which shapes online collegiate networks. The other is the discussion subject. The BBS forum is based on campus life, which brings about plentiful and diverse information of colligate affairs and social issues. It therefore is possible to extract popular topics and their evolution in Chinese universities.

### Data presentation

The data examined in this paper were downloaded from four typical Chinese universities: Peking University (PKU), Renmin University of China (RUC), Shanghai University (SHU) and Fudan University (FDU), where the first two are located in Beijing and the last two are located in Shanghai. All of them are the comprehensive university in China and have big influence among national universities. We download threads from the BBSs to create four sets of data, each of which contains an ensemble of articles. Basic profiles of the data sets are given in S1 Table. From the computer science perspective, the BBS data can be divided into two separate parts: structured and unstructured data. The structured data include post ID, board, time stamp and replied ID. The only unstructured data is text content, usually short and concise, which are written mainly in natural language. This unstructured nature prohibits most conventional data mining techniques from efficacy.

### Users’ preferred boards

**Fig 1:**
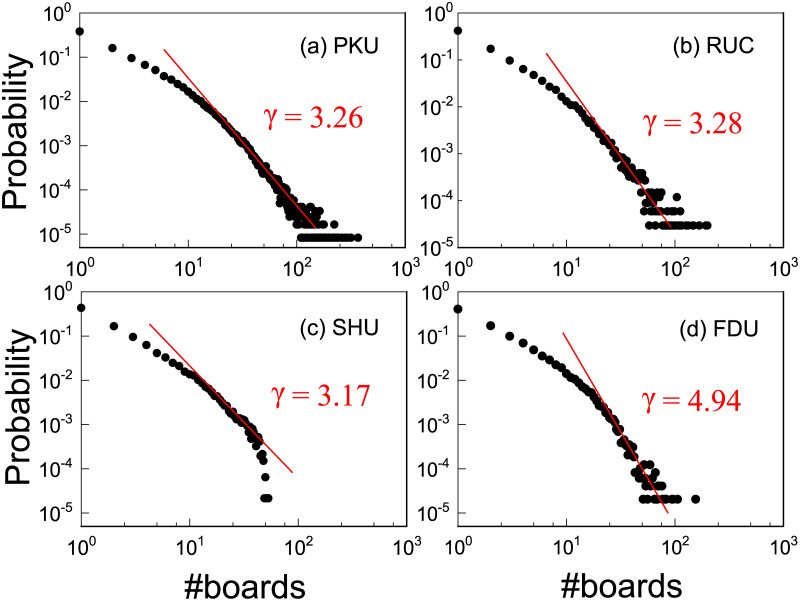
Probability density functions of active boards of users in their lifetime. The red lines are fitted power laws with different values of the exponent *γ*, which are obtained by the likelihood ratio statistical test [22, 23] with *α* < 0.05 for all universities.

Articles in a BBS are posted by users, reflecting their activity. It is well known that online users exhibit great heterogeneity. For example, the lifetime of a user, defined as the time period between the first post and last post, follows a heavy-tailed distribution [9]. During the lifetime, most users don’t stick to one particular board but engages in several boards base on their interests. We compute the distribution of active boards of users in their lifetime for each BBS. As shown in Fig 1, all the plots in the double logarithmic scales are right-skewed and can be fitted by power laws, which indicates that most boards attracted limited attention and were quickly forgotten. On the contrary, a minority of boards became extraordinarily popular among users and acted as core discussion space. To identify users’ preferred boards, we develop a hypothesis-testing method to examine the data. For a certain user, each board with normalized activity *a*_*i*_ has a value

$$\begin{array}{c} p_{i}=\left(m-1\right)\int_{a_{i}}^{1}{\left(1-x\right)}^{m-2}dx={\left(1-a_{i}\right)}^{m-1}, \end{array}$$ (7)

where *m* is the number of boards that the user participates in. Imposing a statistical significance level *α*, we only consider the maximum value of *p*_*i*_; that is, if arg max *p*_*i*_ = *j*, the statistical significant board *j* is defined as the user’s preferred board, which satisfies

$$\begin{array}{c} p_{j}=\underset{1\leq i\leq m}{\text{max}}p_{i}<\alpha . \end{array}$$ (8)

**Fig 2:**
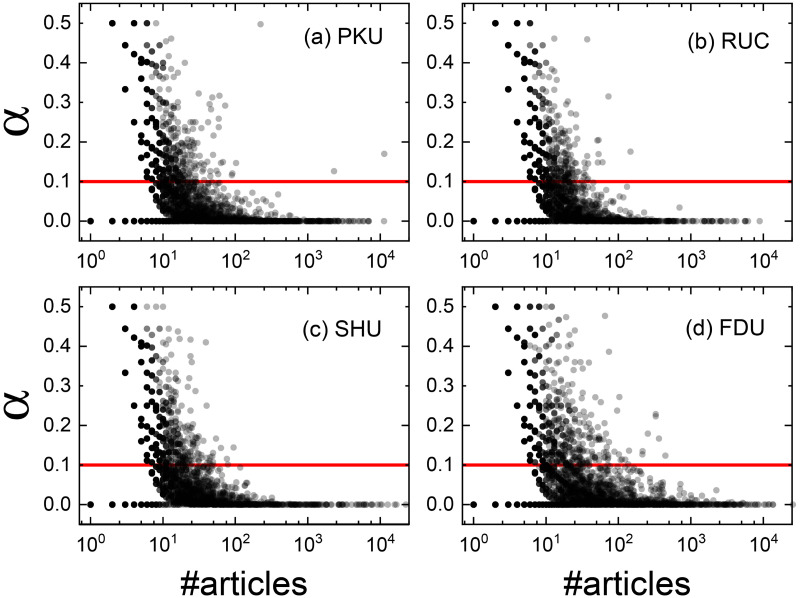
Relation between *p*-value and user activity. The red lines correspond to the significance level *α* = 0.1. More than 70% points are below the line in each university.

In particular, if the user only sticks to one particular board (*m* = 1 in this case), we regard this board as the referred board. It should be stressed that not all users’ preferred boards can be identified, and only those whose maximum board activity satisfies the above criterion can be inferred. Fig 2 presents the relation between users’ *p* values and their action (total number of articles posted by users). Strikingly, users with low or high level of activity exhibit very small values of *p*, implying high possibility to stick to one board. With the significance level *α* = 0.1 (red line), we filter out all users with a uniform selection of boards compatible with the null model. Finally, more than 70% users pass the test whose preferred boards can be inferred. The rest users belong to multi-boards simultaneously. We call them overlapping nodes in the language of the network theory, which have little effect on the boundaries of the resulting community structure. It is interesting to make use of all users’ information to obtain the overlapping community structure. One possible method called collaborative filtering can be processed to characterize all user’s preferences of boards, which can learn the user’s preference vectors automatically. Then, one can apply efficient clustering algorithms on these learned vectors to identify underlying user patterns. However, it is beyond the present study.

### Users’ affiliated communities

**Fig 3:**
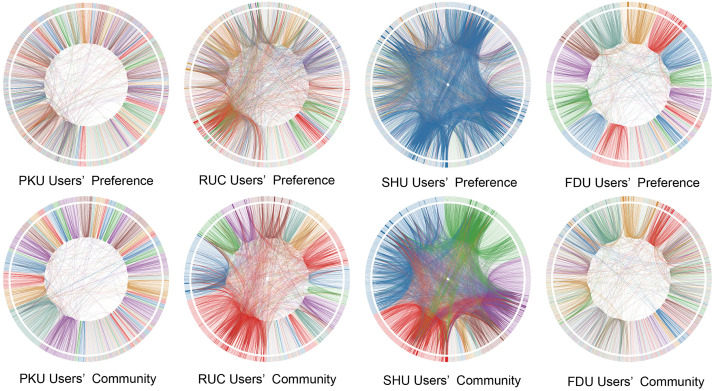
Visual comparison of board-preferred polarizations (upper panel) to algorithmically identified communities (lower panel) for four universities. All the nodes follow the same order in the same university. The colors of the nodes in the upper panel indicate preferred boards, while they indicate communities in the lower panel. The colors of the links is selected randomly from the color of the connected nodes.

We empirically obtain online networks based on replying relationships among users as detailed in the Methods. For each network, a large fraction of nodes are connected, the minimum of which is 83% for RUC, hence the giant component (see S2 Table). Although a wide range of users participate in the discussion in a BBS, few of them show high level of activity, yielding the power-law distribution of nodal degrees (see S2 Fig). Whether users’ tendency to preferred boards elicit clusters? To test this hypothesis, we detect the community structure in the giant component of each university in the same period of 2006-2012 based on a multilevel algorithm [15]. To investigate the correlation between the algorithmically identified community structure and the users’ polarization according to their preferred boards, we compute the Rand index (*S*_R_) [18] and adjusted Rand index (*S*_AR_) [19] to measure the similarity. *S*_AR_ is the corrected-for-chance version of *S*_R_, hence larger discrimination. From Table 1, one can notice that the values of *S*_AR_ for RUC and SHU are much smaller than those for PKU and FDU. To visualize the discrepancy, we present the backbone of each BBS network. As shown in Fig 3 by Circos [24], the outermost circle represents nodes and links in the circle represent the interactions among them. The thickness of a link is proportional to its weight. In the upper panel, different colors indicate users’s different preferred boards, whereas in the lower panel, users follows the same order and different colors represent their memberships in diverse communities. One can see apparent difference between two partitions. So the given categorical boards are inadequate to represent the underlying community structure.

**Table 1:** The Rand index *S*_R_ and adjusted Rand index *S*_AR_ for comparing the community structure of reply networks to the polarization of users according to their preferred boards.

	Connected users	Indicated users	*S*_R_	*S*_AR_
PKU	52,853	37,211	0.84259	0.33748
RUC	22,472	17,344	0.76810	0.01987
SHU	28,315	20,386	0.75714	0.02324
FDU	51,470	38,843	0.96285	0.43294

### Popular topics

**Fig 4:**
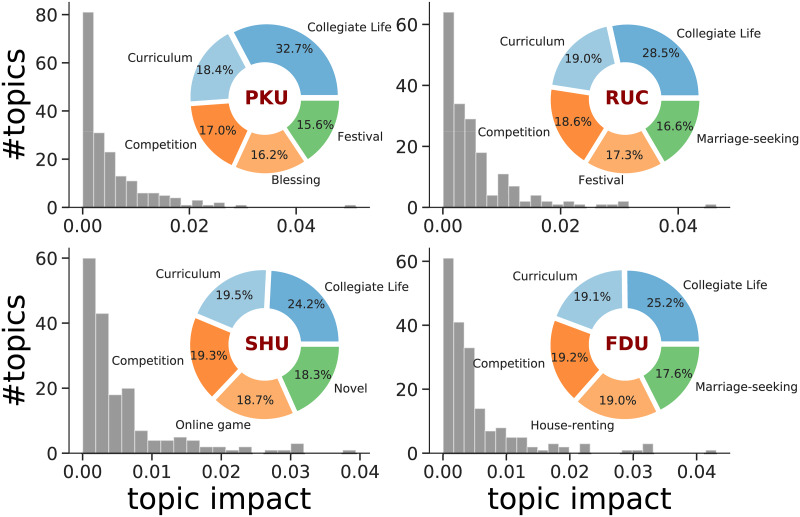
Frequency histograms of topics for four universities around 2006-2012. Each pie chart represents top 5 popular topics in that university with relative impact ratios.

Although a BBS contains hundreds of discussion boards, users usually stick to a minority of them. Thus, popularity information is immersed in the textual content of articles. To find popularity in natural language text documents, we employ LDA methodology [16], which is a popular and powerful topic modeling technique with many applications [25–27]. To compare with different universities, we adopt a novel unified topic modeling framework called Familia [20], which contains well-trained topic models based on various types of large-scale Chinese corpora. We define the textual content of a thread (a collection of articles) as an input document in the LDA framework. The result of applying one input document *d* is a *k*-dimensional topic membership vector

$$\begin{array}{c} \theta_{i}=\left(\theta {\left(d_{i}\right)}_{1},\theta {\left(d_{i}\right)}_{2},\cdots ,\theta {\left(d_{i}\right)}_{k}\right), \end{array}$$ (9)

in which each element represents a *topic impact*. Following Familia, we set *k* = 476 (see Methods for details). For arbitrary *i* and *j*, 0 ≤ *θ*(*d*_*i*_)_*j*_ ≤ 1 and $\sum_{j=1}^{k}\theta {\left(d_{i}\right)}_{j}=1$ always hold. Moreover, each topic is along with hightest-probable words that are semantically related. To reduce the impact of a large number of repeated posts, a thread impact vector is defined as *n*_*i*_ *θ*_*i*_, where *n*_*i*_ denotes the number of users taking part in this thread. Finally, we define the popularity vector with yearly temporal resolution in one university as

$$\begin{array}{c} \Theta_{y}=\frac{1}{N}\sum_{d_{i}\in D\left(y\right)}n_{i}\theta_{i}, \end{array}$$ (10)

where *D*(*y*) is the set of all threads in year *y* and $N=\sum_{i=1}^{\left|D(y)\right|}n_{i}$ is the sum of the number of participants in each thread. So **Θ**_*y*_ measures the distribution over 476 topics in that particular year [28]. We apply this metric to four data sets and find that 100 ∼ 200 topic impacts are positive, the distribution of which is shown in Fig 4, exhibiting heterogeneous impacts. We pick out 5 most influential topics in each university and their relative impact ratio around 2006-2012 (see pie charts in Fig 4). For all universities, the collegiate life is the center of students, while a little difference appers in the 5th topic. One can find further information of each year in supplementary S3 Table.

### Popularity evolution

**Fig 5:**
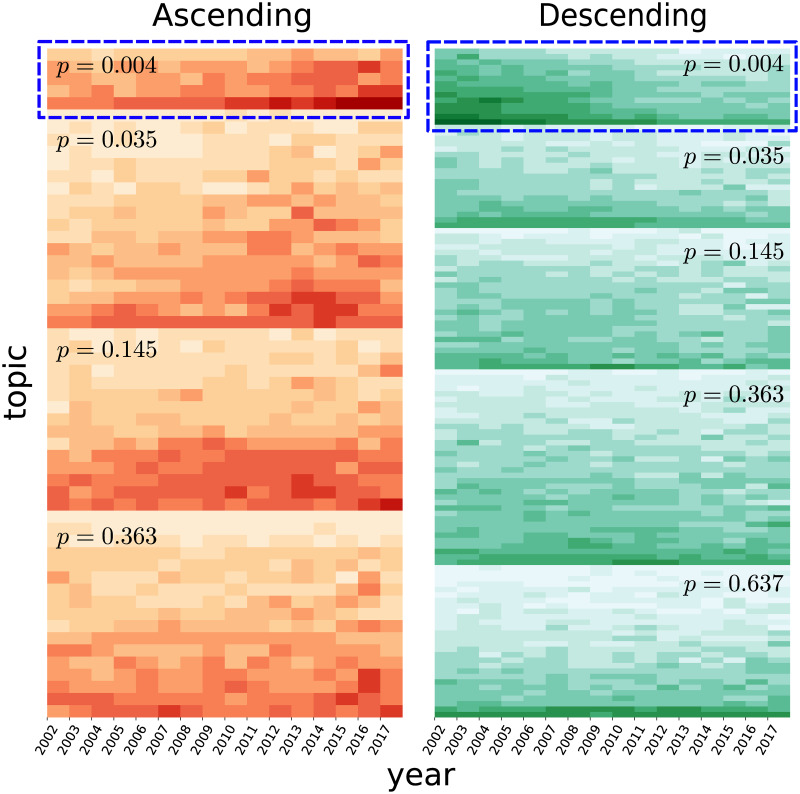
Temporal evolution of topic impacts of PKU around 2002-2017. The Cox-Stuart test divided the topics into ascending (left panel) and descending (right panel) classes, each of which are grouped by *p*-values.

We implement the Cox-Stuart test [17] to quantify the magnitude of the popularity trend of each topic over time. Fig 5 shows the result of PKU as an example. A total of 178 positive topics were obtained, of which 121 topics exhibit the ascending trend (left panel) and 57 topics exhibit the descending trend (right panel). Topics are clustered by their *p*-values and sorted by respective impacts around 2006-2012. The smaller the value of *p* is, the larger impact the topic has. With the minimum *p*-value (box surrounded by blue-dotted line in the left panel), we obtain top 5 ascending topics: *Marriage-seeking*, *House-renting*, *Job recruitment*, *Study overseas*, and *Graduate entrance examination*, which indicate that contemporary college students pay much attention to realistic affairs, such as marriage and job. Meanwhile, pursuing a postgraduate study overseas become popular. For the sake of comparison, we pick out 5 descending topics with larger impacts (box surrounded by blue-dotted line in the right panel), which are *Blessing*, *Literature/Novel*, *Academic conference*, *Online games* and *Show/Art festival*. Interestingly, early students were purer who cared about literature and art, which results in close relationships among them inside the campus, as manifested by greeting each other during festivals. We apply this metric to other three universities and observe similar phenomena (see S3 Fig).

### Simulation of BBS networks

**Fig 6:**
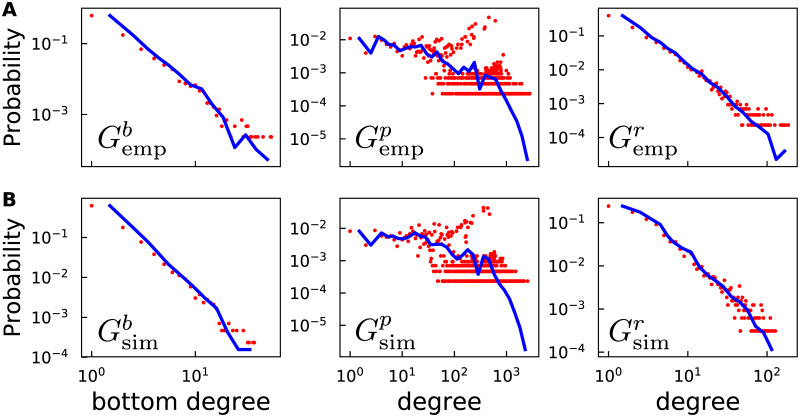
Degree distributions of the bipartite network *G*^*b*^, projection network *G*^*p*^ and reply network *G*^*r*^ based on empirical data (upper panel) and simulated results (lower panel). In our simulation, we set *k* = 2, *μ* = 0.5, *ε* = 0.5, *τ* = 10, and *θ* = 0.028 to generate $G_{\text{sim}}^{r}$. The blue lines are the histograms using logarithmically spaced bins [22].

**Fig 7:**
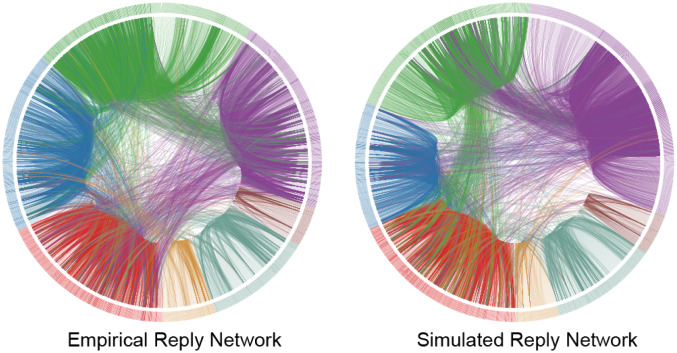
Comparison of the community structure between empirical (left) and simulated (right) networks. The empirical data are taken from PKU in November 2014. The simulated parameters are the same as in Fig 6.

In a BBS, a user chooses certain boards to participate in based on his/her preference and tends to reply to others if they have similar interests. The whole system can be modeled by a bipartite graph *G*^*b*^ = (*B*, *U*, *E*) where *B* denotes boards and *U* denotes users [29]. If a user publishes an article on a board, a link is built between them. The top (board) and bottom (user) degree distributions of empirical bipartite networks $G_{\text{emp}}^{b}$ of the four universities exhibit a striking property (see S4 Fig): bottom degree distributions exhibit power laws while top degree distributions are undeterminable and vary from one to another. This property leads to the following preferential attachment model of the bipartite network $G_{\text{sim}}^{b}$ based on a given top degree distribution *p*(*k*_⊤_). The model starts with an empty graph. At each step, a new top node is added and its degree *k*_⊤_ is sampled from *p*(*k*_⊤_). Then for *k* links of this new top node, either connect to existing bottom nodes via preferential attachment based on their bottom degrees *k*_⊥_ (with probability λ) or connect to a new added bottom node (with probability 1 − λ). By tuning the value of λ one can obtain $G_{\text{sim}}^{b}$ with the same number of top nodes *n*_⊤_ and bottom nodes *n*_⊥_ in $G_{\text{emp}}^{b}$, which yields λ = 0.463 in our simulation. Since our focus is on users, we project $G_{\text{sim}}^{b}$ on users to create the projection graph $G_{\text{sim}}^{p}$ in which nodes represent users and two nodes are connected if both the users post articles on the same board. Fig 6 shows the results of PKU in November 2014 for example. Both $G_{\text{emp}}^{p}$ and $G_{\text{sim}}^{p}$ contain a mass of high-degree nodes, which are derived from board-induced cliques. Both $G_{\text{emp}}^{b}$ and $G_{\text{sim}}^{b}$ contain *n*_⊤_ board-induced cliques *C*_*i*_, *i* = 1, ⋯, *n*_⊤_. Notice that the degree distribution of $G_{\text{emp}}^{p}$ differs from $G_{\text{emp}}^{r}$. This is because not all users participate in the same board and have opportunity to establish a replying relationship. Therefore, we employ a multidimensional bounded confidence model [30], a stochastic model for the evolution of continuous-value opinions, to filtrate links in $G_{\text{emp}}^{p}$ based on *k*-dimensional users’ opinion vector. For each node in $G_{\text{emp}}^{p}$, the initial opinion ***X***(0) ∈ Δ^*k*−1^ (the Δ^*k* − 1^ is (*k* − 1)- Simplex) is sampled from the (*k* − 1)-dimensional uniform distribution. At each time step *t*, for every board-induced cliques *C*, two random users *i*, *j* ∈ *C* are chosen and adjust their opinions according to

$$\begin{array}{c} \begin{array}{c} x_{i}\left(t+1\right)=\{\begin{array}{cc} x_{i}\left(t\right)+\mu \left(x_{j}\left(t\right)-x_{i}\left(t\right)\right), & \;\text{if}\;\frac{1}{k}∥x_{j}\left(t\right)-x_{i}\left(t\right)∥<\varepsilon \\ x_{i}\left(t\right), & \;\text{otherwise}\; \end{array}\\ x_{j}\left(t+1\right)=\{\begin{array}{cc} x_{j}\left(t\right)+\mu \left(x_{i}\left(t\right)-x_{j}\left(t\right)\right), & \;\text{if}\;\frac{1}{k}∥x_{j}\left(t\right)-x_{i}\left(t\right)∥<\varepsilon \\ x_{j}\left(t\right), & \;\text{otherwise}\; \end{array} \end{array} \end{array}$$ (11)

where *μ* is the convergence parameter, *ε* is bounded confidence parameter and ∥.∥ is Euclidean norm. After *τ* iterations, the link will be deleted between two nodes in $G_{\text{sim}}^{p}$ if ∥***x***_*j*_(*τ*) − ***x***_*i*_(*τ*)∥ > *θ*, where *θ* is the tolerance parameter. As shown in Fig 6, for the bipartite network *G*^*b*^, projection network *G*^*p*^ and reply network *G*^*r*^, we notice a good agreement between real data (upper panel) and simulation results (lower panel). In Fig 7, we compare the community structure of empirical reply network $G_{\text{emp}}^{r}$ (left) and simulated reply network $G_{\text{sim}}^{r}$ (right). Different colors correspond to different communities. Again, we see a high level of similarity. Further quantitative information is provided in S5 Fig. Here we adjust our parameter values to fit the real degree distribution of the PKU reply network $G_{\text{emp}}^{r}$. More generally, one can employ the Kullback-Leibler divergence, which is defined by

$$\begin{array}{c} \text{KL}\left(P_{G_{\text{emp}}^{r}}∥P_{G_{\text{sim}}^{r}}\right)=\sum_{k}P_{G_{\text{emp}}^{r}}\left(k\right)\text{log}\frac{P_{G_{\text{emp}}^{r}}\left(k\right)}{P_{G_{\text{sim}}^{r}}\left(k\right)} \end{array}$$ (12)

where $P_{G_{\text{emp}}^{r}}$ is the degree distribution of the empirical reply network and $P_{G_{\text{sim}}^{r}}$ is the degree distribution of the simulated reply network. One obtains appropriate values of the parameters by minimizing the Kullback-Leibler divergence.

## Conclusions

As a new ecosystem of individual interactions, online social networks have become tremendously popular. However, few studies paid attention to Chinese college students. In this article, we have studied online communities (latitudinal property) and popularity (longitudinal property) of BBSs of four Chinese universities. In the community problem, we used the hypothesis test to show that users with low or high level of activity always stick to preferred boards, which yields a polarization. Looking at network communities obtained from empirical reply networks, we found a distinct community structure. Both quantitative and visual tools to measure the similarity between two partitions demonstrated the great discrepancy, indicating that board labels are inadequate to represent underlying communities. The observed structure can be reproduced by a simple model that mimics the preferential interests of users. In the complementary problem of popularity, we developed LDA methodology to discover topics from text corpora, which allows us to compare popularity in different universities. Based on the Cox-Stuart test, we extracted ascending and descending topics around a decade. The significant trendlines imply that contemporary students in Chinese universities pay much attention to marriage, job and postgraduate study compared with earlier ones. These results illustrate how latitudinal and longitudinal perspectives give complementary insights on social life in Chinese universities, and might shed light in understanding adolescent society in China.

## Supporting information

S1 Fig The hierarchical structure of a BBS (a) and a typical reply article (b). (PDF)

S2 Fig Degree distributions of reply networks with fitted power laws. (PDF)

S3 Fig Top increasing and decreasing topics in each university. (PDF)

S4 Fig Top and bottom degree distributions of empirical bipartite network in each university. (PDF)

S5 Fig Community size of the simulated model and empirical network. (PDF)

S1 Table Characteristics of the four data sets in present study. (PDF)

S2 Table The largest connected components of the four BBS time-aggregated reply networks among 2006-2012. (PDF)

S3 Table Top five hotest topics in different universities among 2006-2012. (PDF)
