# Supplementary figures and images for: A comparative study of online communities and popularity of BBS in four Chinese universities
Source: PLoS One. 2020 Jun 24;15(6):e0234469. doi: 10.1371/journal.pone.0234469 (PMC7313755; doi:10.1371/journal.pone.0234469)

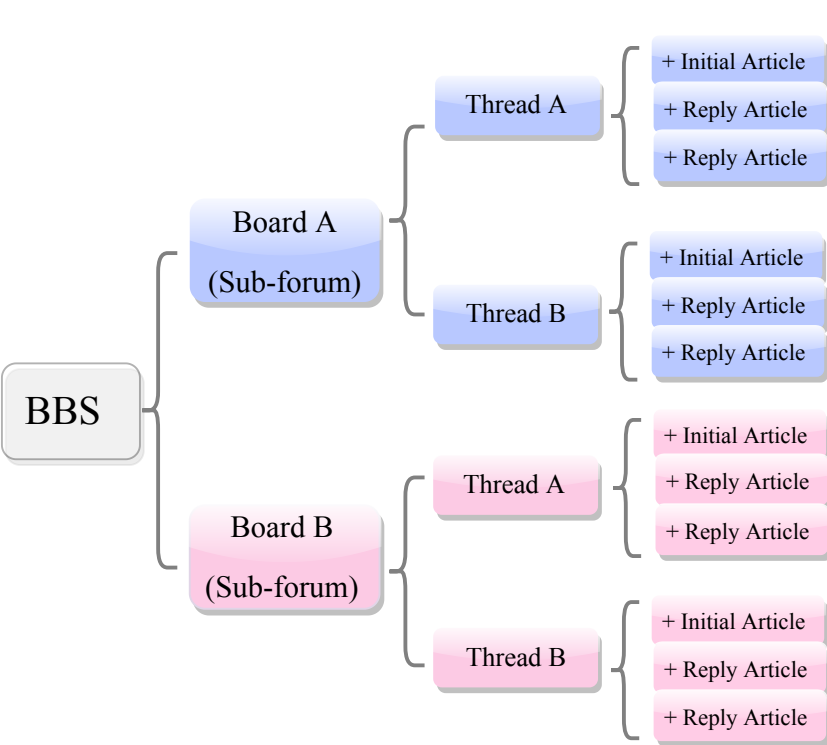

(a)

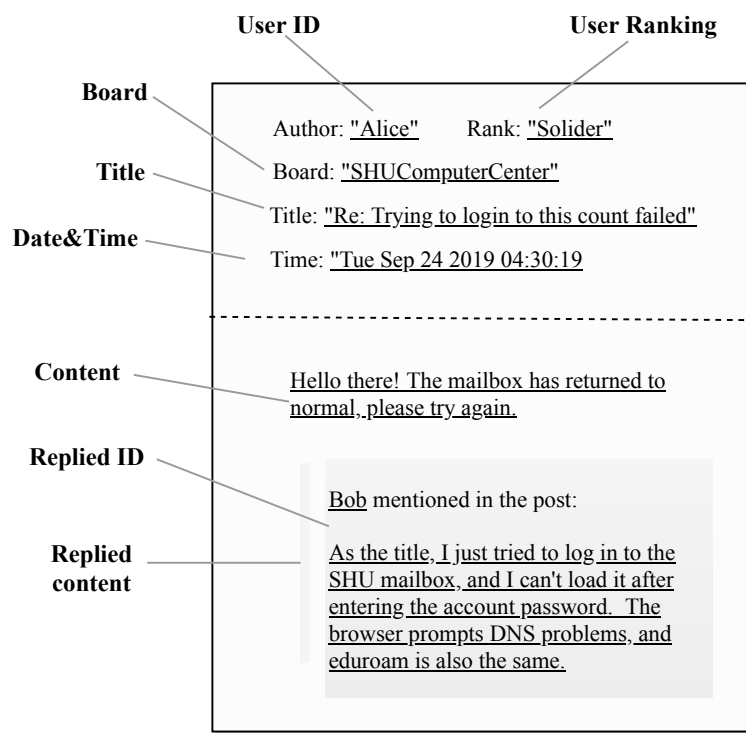

(b)

Supplement: S1 Fig — (PDF) [file pone.0234469.s001.pdf]

PDF

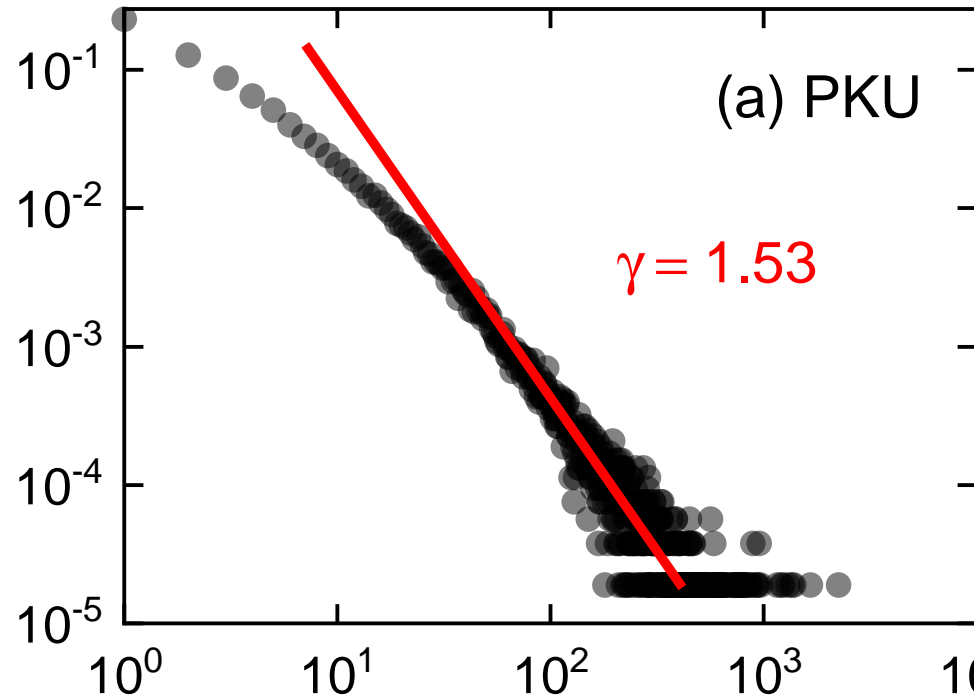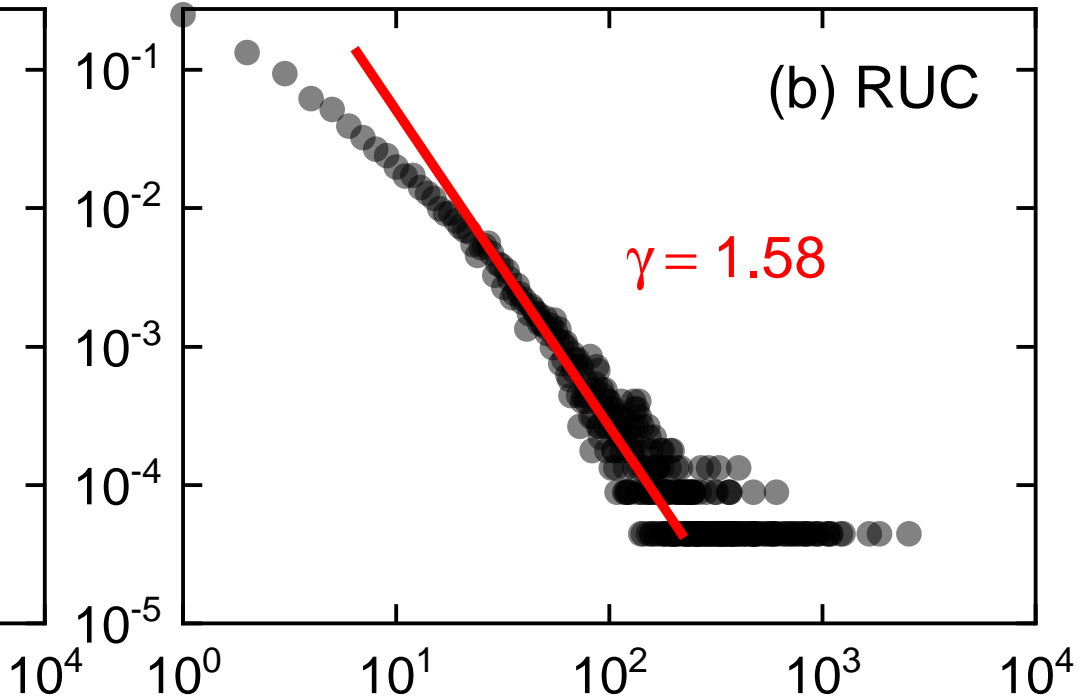

PDF

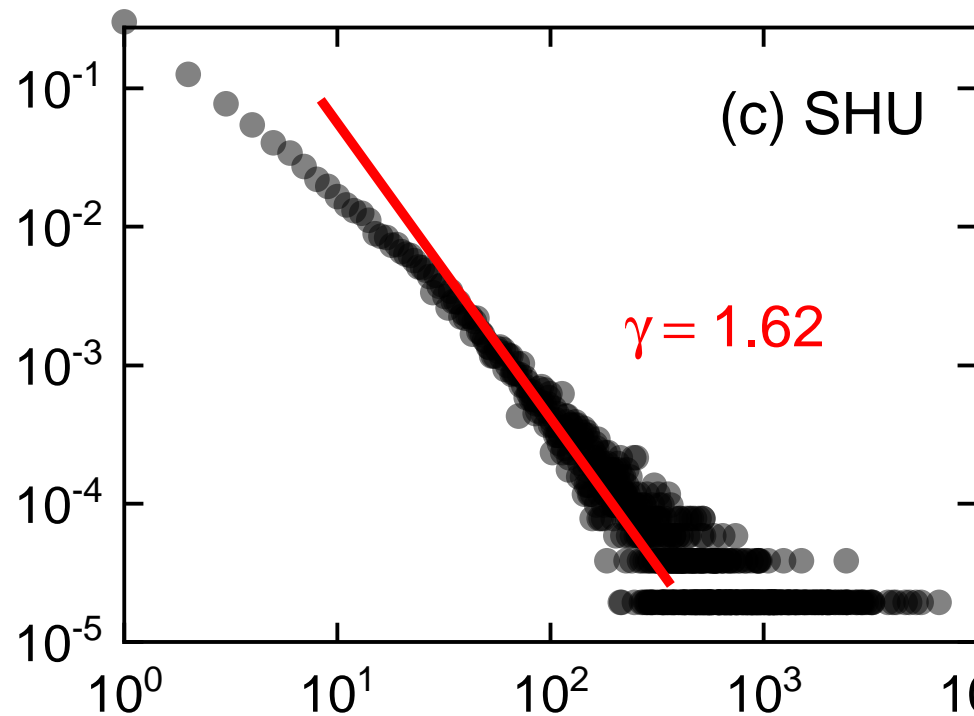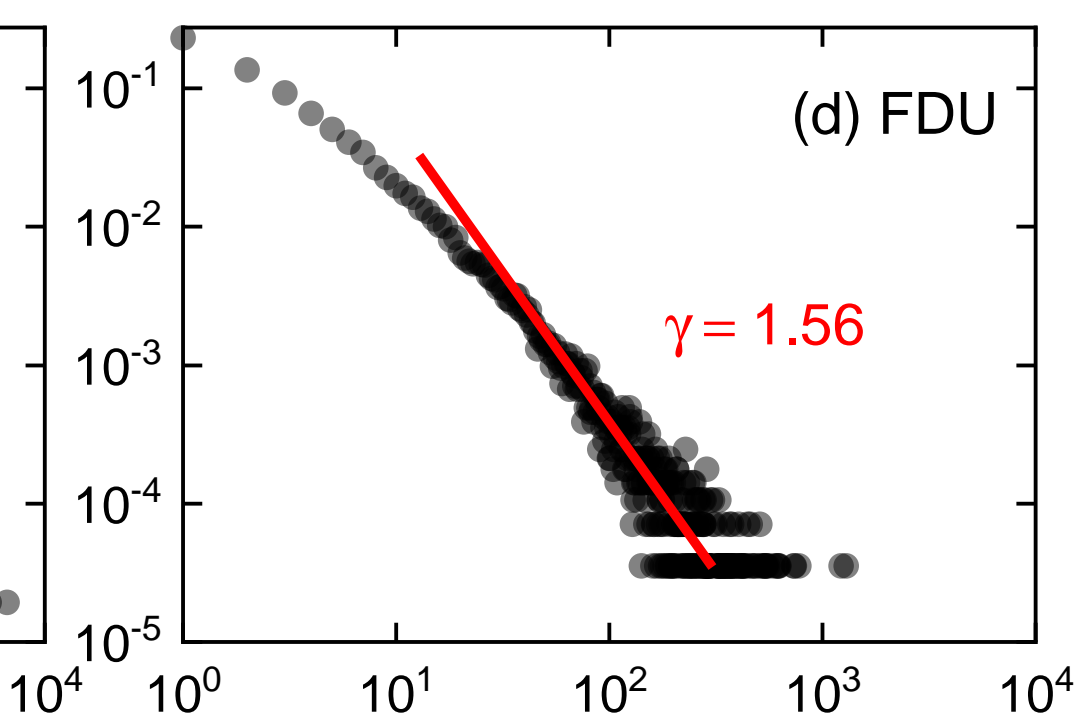

degree

degree

Supplement: S2 Fig — (PDF) [file pone.0234469.s002.pdf]

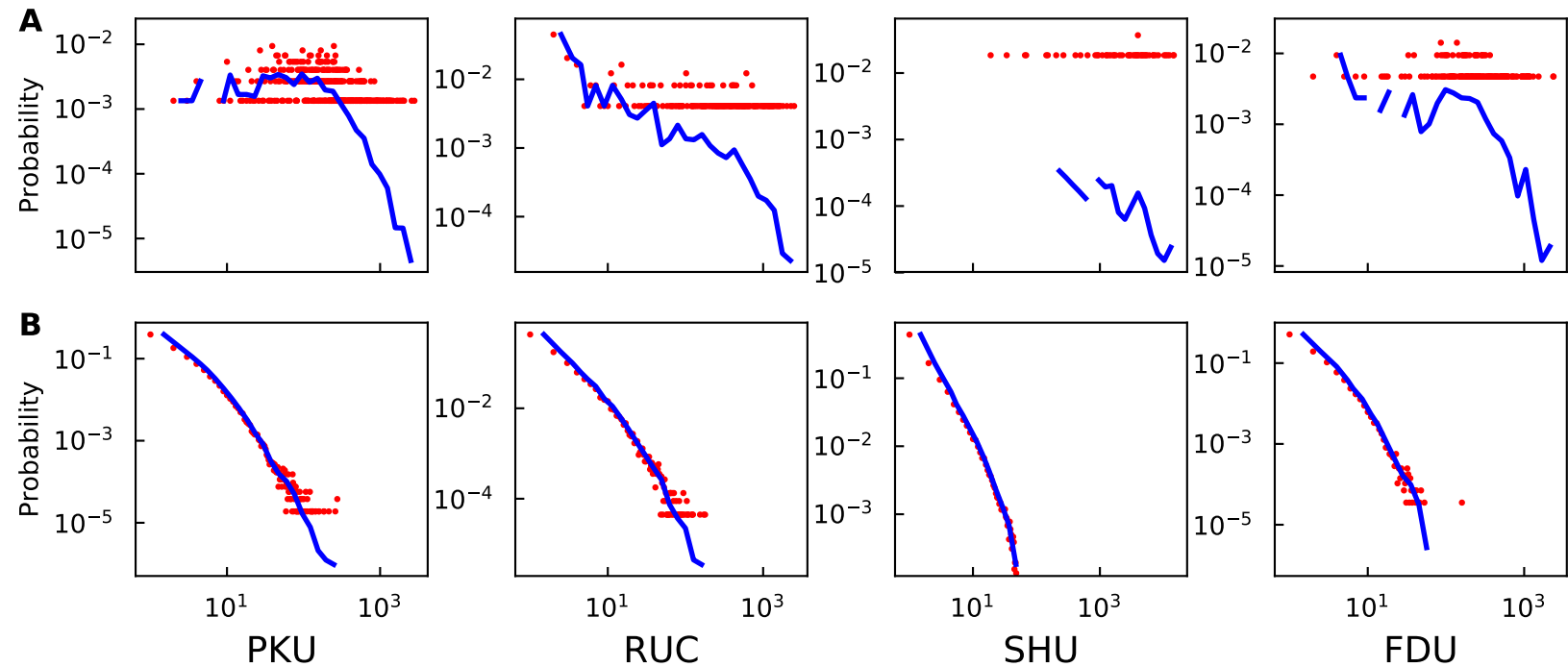

Supplement: S4 Fig — (PDF) [file pone.0234469.s004.pdf]

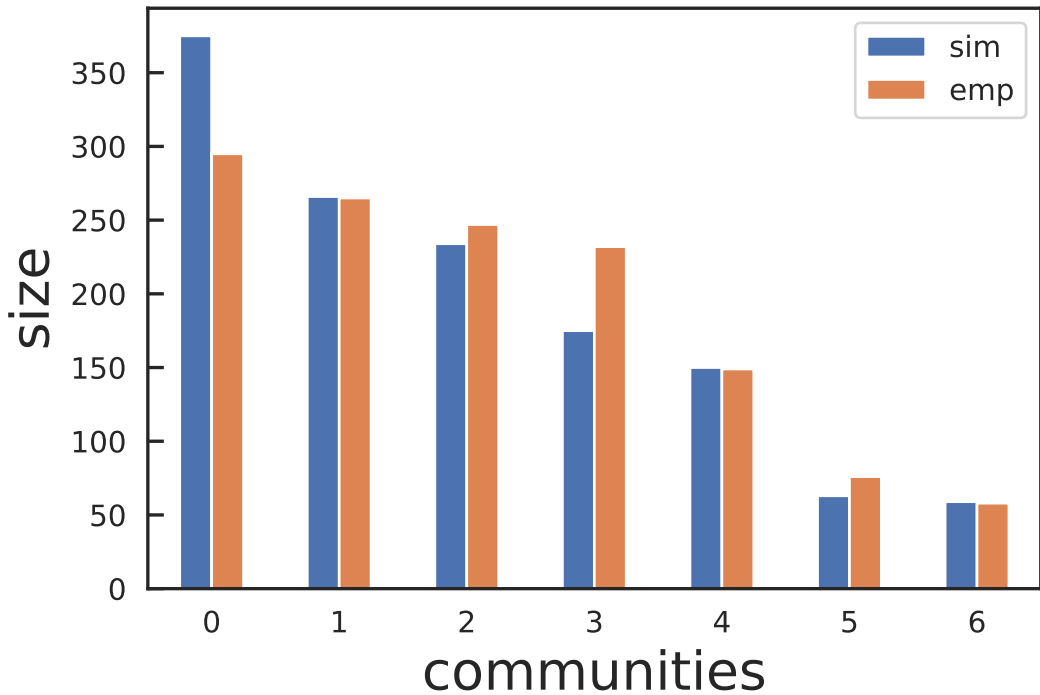

Supplement: S5 Fig — (PDF) [file pone.0234469.s005.pdf]
