# Supplementary material for: A comparative study of online communities and popularity of BBS in four Chinese universities
Source: PLoS One. 2020 Jun 24;15(6):e0234469. doi: 10.1371/journal.pone.0234469 (PMC7313755; doi:10.1371/journal.pone.0234469)
Supplement: S3 Fig — (PDF) [file pone.0234469.s003.pdf]

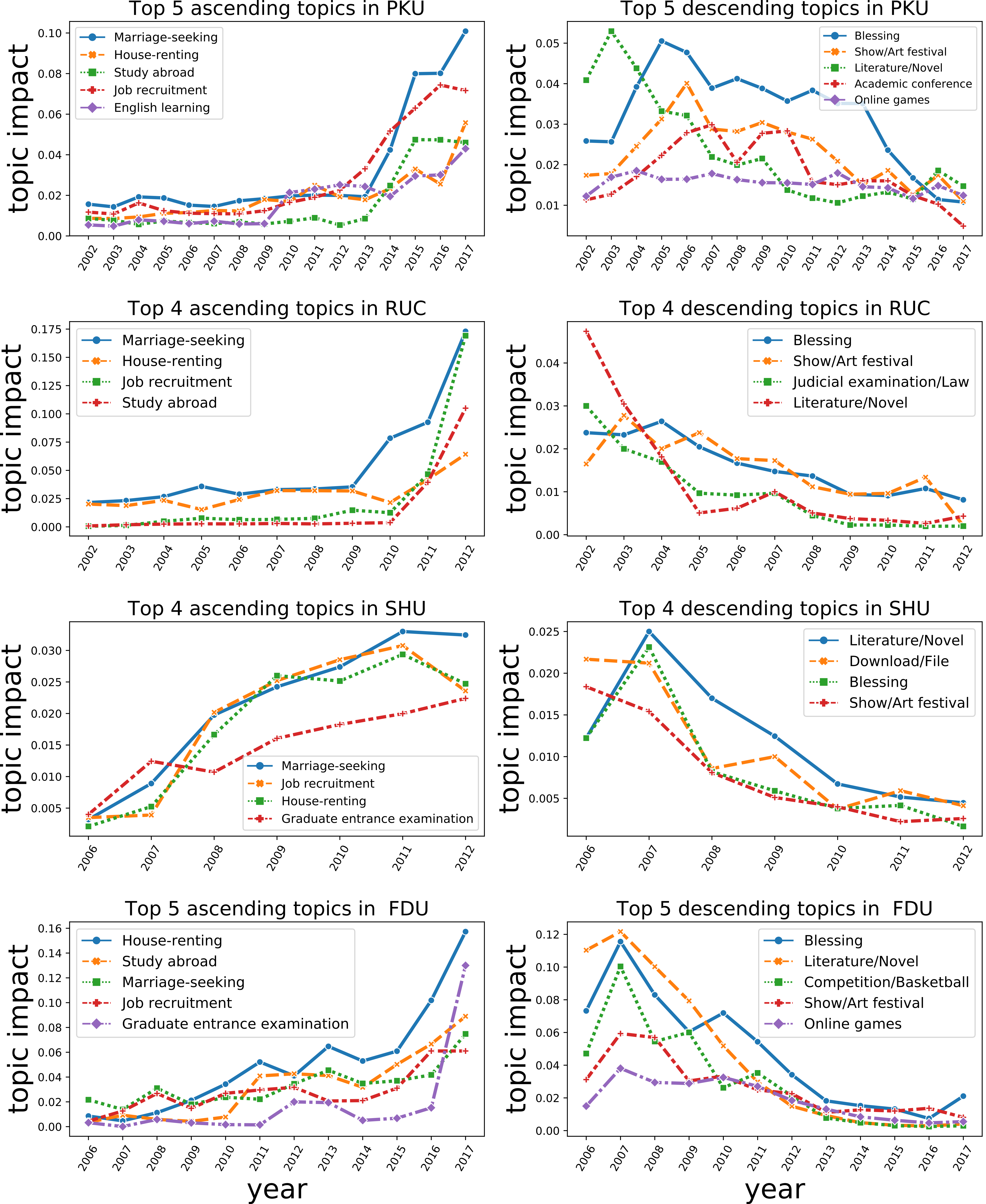
Top 5 ascending topics in PKU
Marriage-seeking
House-renting
Study abroad
Job recruitment
English learning
Top 5 descending topics in PKU
Blessing
Show/Art festival
Literature/Novel
Academic conference
Online games
Top 4 ascending topics in RUC
Marriage-seeking
House-renting
Job recruitment
Study abroad
Top 4 descending topics in RUC
Blessing
Show/Art festival
Judicial examination/Law
Literature/Novel
Top 4 ascending topics in SHU
Marriage-seeking
Job recruitment
House-renting
Graduate entrance examination
Top 4 descending topics in SHU
Literature/Novel
Download/File
Blessing
Show/Art festival
Top 5 ascending topics in FDU
House-renting
Study abroad
Marriage-seeking
Job recruitment
Graduate entrance examination
Top 5 descending topics in FDU
Blessing
Literature/Novel
Competition/Basketball
Show/Art festival
Online games
topic impact
year
