# Supplementary material for: A comparative study of online communities and popularity of BBS in four Chinese universities
Source: PLoS One. 2020 Jun 24;15(6):e0234469. doi: 10.1371/journal.pone.0234469 (PMC7313755; doi:10.1371/journal.pone.0234469)
Supplement: S1 Table — (PDF) [file pone.0234469.s006.pdf]

	PKU	RUC	SHU	FDU
Period	2002-2017	2002-2012	2006-2012	2006-2017
Users	126,347	34,257	51,738	49,948
Boards	796	308	54	233
Threads	1,537,640	263,551	175,273	420,233
Articles	5,195,305	875,812	2,741,010	3,289,315
