# Supplementary material for: A comparative study of online communities and popularity of BBS in four Chinese universities
Source: PLoS One. 2020 Jun 24;15(6):e0234469. doi: 10.1371/journal.pone.0234469 (PMC7313755; doi:10.1371/journal.pone.0234469)
Supplement: S2 Table — (PDF) [file pone.0234469.s007.pdf]

Institution	PKU	RUC	FDU	SHU
Nodes	62,846	27,069	33,038	53,738
Connected nodes	52,853	22,472	28,315	51,470
Connected edges	437,988	167,113	211,613	644,873
Clustering coefficient	0.1610	0.1612	0.1947	0.2407
Mean degree	16.6	14.8	14.9	25.1
communities	37	20	17	8
Modularity	0.5574	0.4018	0.6150	0.3914
