# Supplementary material for: A comparative study of online communities and popularity of BBS in four Chinese universities
Source: PLoS One. 2020 Jun 24;15(6):e0234469. doi: 10.1371/journal.pone.0234469 (PMC7313755; doi:10.1371/journal.pone.0234469)
Supplement: S3 Table — (PDF) [file pone.0234469.s008.pdf]

	PKU	RUC	SHU	FDU
2006	Competition (0.055)<br>Collegiate life (0.053)<br>Blessing (0.048)<br>Making friends (0.042)<br>Curriculum (0.036)	Collegiate life (0.049)<br>Curriculum (0.047)<br>Making friends (0.035)<br>Competition (0.028)<br>Show/Art Festival (0.023)	Collegiate life (0.054)<br>Making friends (0.048)<br>Curriculum (0.033)<br>BBS regulation (0.028)<br>Download/File (0.022)	Literature/Novel (0.117)<br>Blessing (0.073)<br>Competition (0.048)<br>Curriculum (0.045)<br>Collegiate life (0.030)
2007	Collegiate life (0.058)<br>Competition (0.051)<br>Blessing (0.048)<br>Curriculum (0.037)<br>Show/Art Festival (0.028)	Collegiate life (0.051)<br>Curriculum (0.048)<br>Making friends (0.033)<br>Competition (0.031)<br>Marriage-seeking (0.025)	Collegiate life (0.051)<br>Making friends (0.049)<br>Curriculum (0.041)<br>BBS regulation (0.026)<br>Literature/Novel (0.025)	Literature/Novel (0.120)<br>Blessing (0.115)<br>Competition (0.100)<br>Show/Festival (0.059)<br>Curriculum (0.043)
2008	Collegiate life (0.052)<br>Competition (0.052)<br>Curriculum (0.044)<br>Blessing (0.040)<br>Olympic games (0.022)	Collegiate life (0.048)<br>Curriculum (0.044)<br>Making friends (0.039)<br>Competition (0.025)<br>Blessing (0.015)	Collegiate life (0.047)<br>Making friends (0.043)<br>Curriculum (0.040)<br>BBS regulation (0.021)<br>Job recruitment (0.020)	Literature/Novel (0.090)<br>Blessing (0.081)<br>Competition (0.073)<br>Show/Festival (0.057)<br>Curriculum (0.057)
2009	Collegiate life (0.051)<br>Competition (0.050)<br>Curriculum (0.044)<br>Blessing (0.040)<br>Marriage-seeking (0.018)	Collegiate life (0.048)<br>Curriculum (0.044)<br>Entertainment (0.039)<br>Competition (0.037)<br>Blessing (0.018)	Collegiate life (0.052)<br>Making friends (0.040)<br>Curriculum (0.040)<br>House-renting (0.025)<br>Job recruitment (0.024)	Literature/Novel (0.081)<br>Blessing (0.061)<br>Competition (0.061)<br>Curriculum (0.045)<br>Collegiate life (0.025)
2010	Collegiate life (0.059)<br>Competition (0.054)<br>Curriculum (0.044)<br>Blessing (0.038)<br>English learning (0.022)	Marriage-seeking(0.075)<br>Collegiate life (0.044)<br>Curriculum (0.039)<br>Competition (0.022)<br>House-renting (0.023)	Collegiate life (0.045)<br>Entertainment (0.039)<br>Curriculum (0.038)<br>Job recruitment (0.026)<br>Marriage-seeking (0.025)	Blessing (0.071)<br>Literature/Novel (0.081)<br>Competition (0.052)<br>Curriculum (0.051)<br>Collegiate life (0.033)
2011	Collegiate life (0.057)<br>Competition (0.052)<br>Curriculum (0.048)<br>Blessing (0.038)<br>House-renting (0.022)	Marriage-seeking(0.083)<br>Collegiate life (0.044)<br>Curriculum (0.039)<br>Job recruitment (0.035)<br>Competition (0.0028)	Collegiate life (0.041)<br>Marriage-seeking (0.035)<br>Curriculum (0.032)<br>Job recruitment (0.028)<br>House-renting (0.027)	House-renting (0.052)<br>Blessing (0.053)<br>Competition (0.053)<br>Curriculum (0.048)<br>Study oversea (0.033)
2012	Collegiate life (0.053)<br>Competition (0.055)<br>Curriculum (0.050)<br>English learning (0.032)<br>House-renting (0.025)	Marriage-seeking(0.163)<br>Job recruitment (0.162)<br>Study oversea (0.102)<br>Collegiate life (0.055)<br>House-renting (0.053)	Collegiate life (0.044)<br>Marriage-seeking (0.034)<br>Curriculum (0.032)<br>House-renting (0.026)<br>Job recruitment (0.020)	Collegiate life (0.052)<br>Study oversea (0.051)<br>Competition (0.050)<br>Curriculum (0.049)<br>House-renting (0.044)
